# Frequency-Dependent Effects of Material Extrusion Parameters on the Storage Modulus and Loss Factor of PETG

**DOI:** 10.3390/polym18111412

**Published:** 2026-06-05

**Authors:** Sven Gerdes, Philipp M. Heck, Sabine C. Langer, Thomas Vietor

**Affiliations:** 1 Institute for Engineering Design, Technische Universität Braunschweig, 38108 Brunswick, Germany; t.vietor@tu-braunschweig.de; 2 Institute for Acoustics and Dynamics, Technische Universität Braunschweig, 38106 Brunswick, Germany; philipp.heck@tu-braunschweig.de (P.M.H.); s.langer@tu-braunschweig.de (S.C.L.)

**Keywords:** material extrusion, additive manufacturing, dynamic mechanical analysis, process parameters, frequency-dependent mechanical properties

## Abstract

Additive manufacturing by material extrusion enables the fabrication of geometrically complex components, yet the extent to which process parameters can be used to tailor stiffness and damping in a frequency-dependent manner remains insufficiently understood. This study investigates the influence of key material-extrusion process parameters (layer height, printing speed, extrusion temperature, build plate temperature, and flow rate) on the flexural storage modulus $E^{\prime }$ and loss factor η of PETG specimens over a frequency range of 125 to 4000 Hz. Frequency-resolved regression models were established for six reference frequencies using VIF-based term reduction and hierarchical backward elimination. The results reveal a clear contrast between stiffness- and damping-related responses. The model structure for $E^{\prime }$ remained invariant across all frequencies, achieving consistently high coefficients of determination ($R^{2}$ = 0.831–0.847). In contrast, the model structure for η varied markedly with frequency ($R^{2}$ = 0.215–0.763). Extrusion temperature was identified as a consistently significant factor for η across all frequencies (p<0.05), while a robust nonlinear dependence on flow rate dominated most frequency bands. Reduced model adequacy for η was observed at specific bands, showing significant lack-of-fit at 500 Hz ($p_{LOF}=0.049$) and non-normal residuals at 4000 Hz ($p_{JB}=0.003$). These findings demonstrate that stiffness can be tuned reliably using frequency-invariant process relationships, whereas damping requires frequency-aware parameter selection. This approach provides a statistically rigorous basis for optimizing additively manufactured components where both stiffness and energy dissipation are performance-critical.

## 1. Introduction

Material extrusion-based additive manufacturing (MEX) has become one of the most widely used additive manufacturing technologies for polymer components due to its geometric flexibility, material efficiency, and cost-effectiveness [1,2]. Beyond rapid prototyping, MEX is increasingly employed for functional and load-bearing parts in engineering applications, where mechanical performance represents a critical design criterion [2]. Consequently, understanding how process parameters influence the mechanical behavior of MEX-produced components remains a central research topic [3].

The mechanical properties of MEX-produced parts are substantially influenced by process-induced microstructural features, including inter-filament bonding quality, porosity, and geometric discretization effects resulting from the layer-wise deposition process [4]. Consequently, numerous studies have investigated the influence of key process parameters, such as layer height, printing speed, extrusion temperature, build plate temperature, and flow rate, on the mechanical performance of MEX components [3,5]. The majority of this existing work focuses on static or quasi-static mechanical properties, which are typically evaluated using tensile, compressive, or flexural tests conducted at a single loading rate [3].

Frequency-dependent stiffness and damping characteristics are particularly relevant for lightweight MEX structures subjected to dynamic excitation, where the vibration response is governed not only by static stiffness but also by viscoelastic energy dissipation. Typical application examples include drone frames, machine tool holders, and automotive interior components, in which insufficient damping performance may result in elevated vibration amplitudes and acoustic emissions. Dynamic mechanical properties are commonly assessed using dynamic mechanical analysis (DMA) or vibration-based methods, as standardized in ISO 6721 [6,7] and discussed in the established literature [8]. DMA enables the separation of the mechanical response into a storage modulus $E^{\prime }$, representing elastic energy storage, and a loss factor η, quantifying energy dissipation [8].

Recent studies have demonstrated pronounced frequency-dependent viscoelastic behavior in additively manufactured polymers [9,10,11], and dynamic characterization approaches have also been applied to multi-material polymer designs [12]. Furthermore, while the frequency-dependent storage modulus and stress relaxation of PETG-based composites have been characterized in bulk form across a range of 0.1–150 Hz [13], these findings are not directly transferable to MEX components. The mechanical response in MEX is fundamentally governed by process-specific factors like inter-filament bonding and porosity, which are absent in bulk material studies. Nevertheless, systematic investigations that quantitatively link MEX process parameters to frequency-dependent mechanical properties across a broad frequency range using statistically rigorous modeling approaches remain scarce compared to the extensive body of literature on static and quasi-static behavior [3,5]. A recent example of such quasi-static optimization is the work of Ulkir et al. [14], who applied a Taguchi L27 design and ANOVA to investigate the influence of MEX process parameters on the tensile strength of PA12-CF composites. Their results identified infill density as the dominant factor governing tensile strength. However, it remains unclear whether the same parameter sensitivities persist under dynamic loading conditions, where viscoelastic energy storage and dissipation mechanisms become relevant.

Moreover, process parameter effects on stiffness-related and dissipation-related responses are frequently analyzed jointly or reduced to averaged metrics, potentially obscuring differences in their underlying sensitivities. Given that elastic stiffness and damping originate from distinct physical mechanisms, it cannot be assumed a priori that both responses are governed by the same set of process parameters or exhibit similar robustness with respect to excitation frequency [8]. Addressing this gap requires an experimental and statistical framework capable of resolving parameter effects as a function of frequency while maintaining model interpretability and robustness.

In this study, a systematic design-of-experiments approach is combined with frequency-resolved regression modeling to investigate the influence of key MEX process parameters on the flexural storage modulus $E^{\prime }$ and the loss factor η of additively manufactured specimens. Separate regression models are constructed for multiple discrete frequency bands, enabling a direct comparison between frequency-invariant and frequency-dependent process sensitivities. Model complexity is controlled through variance inflation factor-based term reduction, hierarchical backward elimination, and comprehensive diagnostic assessment, including residual analysis and lack-of-fit testing.

To address the identified lack of statistically rigorous models linking manufacturing settings to dynamic performance, the objectives of this work are threefold: (i) to identify which process parameters robustly govern stiffness-related responses across frequencies, (ii) to determine how dissipation-related responses depend on frequency and parameter coupling, and (iii) to assess the adequacy of polynomial regression models for capturing frequency-dependent behavior in MEX materials. This is achieved by combining a systematic design-of-experiments approach with frequency-resolved regression modeling and comprehensive diagnostic assessments. By explicitly distinguishing between storage- and loss-dominated process sensitivities within a unified framework, this study provides new insights into the frequency-dependent process–structure–property relationships of MEX components and establishes a robust basis for process optimization in vibration-critical engineering applications.

## 2. Materials and Methods

This section describes the materials, manufacturing equipment, and experimental methods used for specimen fabrication and subsequent characterization.

### 2.1. Materials

All specimens were manufactured from a commercial PETG filament (PETG Filament–1.75 mm–Black–1 kg, Das Filament, Emskirchen, Germany) with a nominal diameter of 1.75 mm. PETG is a glycol-modified copolymer of polyethylene terephthalate that provides good interlayer adhesion, low shrinkage, and mechanical robustness, making it suitable for the MEX processes. Prior to processing, the filament was dried in an oven (ULM 400, MEMMERT GmbH, Schwabach, Germany) at 70 °C for 12 h to reduce moisture content and moisture-related printing defects.

### 2.2. Specimen Fabrication

Specimens were fabricated using a MEX 3D printer (Funmat Pro 410, INTAMSYS, Shanghai, China) equipped with a ceramic glass build plate and a nozzle diameter of 0.4 mm. Prior to printing, the build plate was coated with an adhesion promoter (3DLAC, Laboratorios Belloch, Zamora, Spain) to ensure reliable first-layer adhesion. All specimens were manufactured under controlled laboratory conditions, with the printer enclosure lid kept open during fabrication in order to avoid heat accumulation. The printing parameters were defined according to a factorial design of experiments and systematically varied as described in Section 2.3. Unless stated otherwise, specimens were printed in a flat orientation on the build platform.

All specimens were manufactured with identical nominal dimensions of 200 mm × 10 mm × 3 mm. Each specimen was printed with two outer perimeters. The internal structure was generated using an alternating ±45° infill pattern with 100% infill density, which was applied consistently throughout the entire specimen height. No variations in process parameters were introduced between individual layers and all layers were fabricated using identical printing conditions. Specimens were manufactured in batches of four and produced sequentially within each batch. Within a given batch, all specimens shared the same layer height, extrusion temperature, and build plate temperature. In contrast, printing speed and flow rate were allowed to vary between specimens within a batch.

After printing, the specimens were cooled to room temperature on the build plate and removed without any additional post-processing.

### 2.3. Experimental Design

The investigated input factors comprised layer height, printing speed, extrusion temperature, build plate temperature, and flow rate, while all remaining process parameters were kept constant. Each factor was examined at three levels using a face-centered composite design (FCCD) to systematically assess the individual parameter effects. The minimum and maximum levels of each factor were selected such that reliable fabrication of the specimens was just achievable, thereby defining a process window that reflects the practical limits of stable printing. A FCCD was employed to ensure specimen manufacturability while maintaining systematic coverage of the investigated parameter space. The described experimental design resulted in the fabrication of 46 specimens (Table 1).

Based on the initial FCCD analysis, which revealed significant quadratic effects for the primary process parameters, a second experimental block was conducted to further resolve these nonlinearities. The experimental design was expanded to five levels using a systematic one-factor-at-a-time extension strategy originating from the design center. To achieve this, intermediate settings at the coded levels of −0.5 and +0.5 were introduced for layer height, printing speed, and flowrate. To isolate the influence of these variables, printing temperature and build plate temperature were kept constant at 237.5 °C and 75 °C, respectively. Each configuration was manufactured in duplicate to ensure statistical reproducibility and to account for production-related variances. The resulting 30 specimens were integrated into the original dataset to augment the regression model and enhance the resolution of the identified quadratic response surfaces.

### 2.4. Method for Experimentally Determining the Viscoelastic Properties of PETG

**Figure 1:**
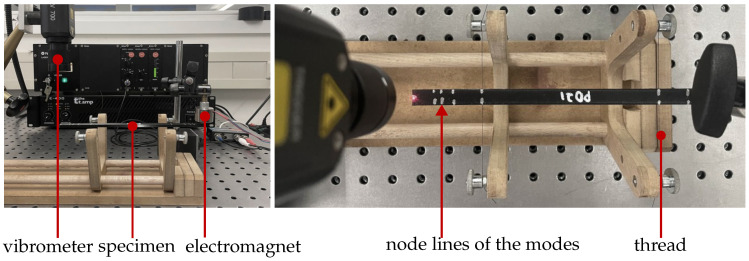
Representation of the experimental setup used. (**Left**) Side view with the measurement technology used; (**right**) Top view of the sample suspension in the first node line.

To determine the dynamic mechanical properties of PETG, the complex flexural modulus $\underline{E}=E^{\prime }\left(1+j\eta \right)$ is determined experimentally; $E^{\prime }$ denotes the flexural storage modulus and η the loss factor. The flexural modulus $E^{\prime }$ is proportional to the maximum stored energy over a load cycle, while the flexural loss modulus $E^{\prime }\eta$ is proportional to the dissipated energy over a load cycle [6]. For the experimental determination of viscoelastic properties, an adapted version of method B (see Figure 1) of the standardised resonance curve method (ISO 6721-3) is used [7]. The need for adjustment arises from the fact that the Euler–Bernoulli beam theory is used in the standard for determining the flexural storage modulus. With increasing mode number and deviating validity of the model assumptions due to geometric dimensions, the deviations in the determination of material parameters increase, as rotational inertia and shear deformations are not taken into account. Furthermore, the boundary conditions are not fully represented, as a small metal plate is glued to the plastic sample (see Figure 1) for contactless excitation by means of electromagnets in order to transmit the excitation forces. By applying Timoshenko’s beam theory and taking into account the inertial force of the additional mass, the flexural modulus of the PETG samples can be determined more precisely [12]. Instead of the method according to 3 dB for determining the loss factor η according to the standard, a frequency response approximation method based on a modal equivalence model according to VDI 3830 Part 5 is used [15].

Based on the measuring principle of the resonance curve method in accordance with ISO 6721-3, the beam is excited at one end by an electromagnet and the dynamic behaviour of the beam is measured at the other end using a laser vibrometer. Since the standardised measurement method is a resonance-based method, the viscoelastic material properties can only be evaluated at the resonance points of the beam. Since every dynamic system exhibits characteristic natural vibration behaviour depending on the boundary conditions, the beam is supported at its respective outer node lines (see Figure 1) in order to satisfy the assumption of a free-free beam. In order to minimise the measurement effort for the large-scale experimental study and at the same time obtain a sufficient number of reference points for the material parameters in the frequency range, the test samples are suspended exclusively in the node lines of the first four bending modes. It is assumed that the influence of incorrect storage in the higher modes of vibration is of minor importance, as investigated in a study by Rotermund et al. [9]. The calculated material properties above the fourth mode order then correspond to the mean values of the respective four measurements.

For the experimental determination of the dynamic behaviour of the different beam samples, a compact laser scanning vibrometer from Polytec (Polytec GmbH, Waldbronn, Germany) with a CLV-700 head and a CLV-1000 controller with a sensitivity of 5 mms^−1^ V^−1^ is used to measure the surface velocity. A 16-bit Triton USB audio interface from Acoustics Engineering (Acoustics Engineering, Boxmeer, The Netherlands) with a sampling frequency of 48 kHz is used for signal processing. The dynamic excitation is performed with a linear sine sweep in a frequency range from 20 Hz to 5 kHz with a total measurement duration of 3 s.

**Figure 2:**
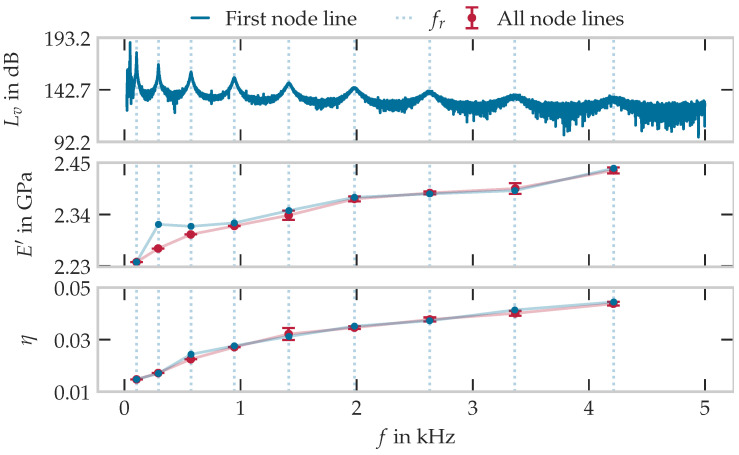
Exemplary representation of the dynamic behaviour and the resulting material parameters of a beam sample. (**Top**) Level of surface velocity, (**centre**) Flexural storage modules determined from the suspension in the first node line (blue) and from all node lines (red), (**bottom**) Lossfactor determined from the suspension in the first node line (blue) and from all node lines (red).

Figure 2 shows an example of the results of a sample beam suspended in the first node line and the resulting material parameters from the four measurements with the respective specific suspension position. The surface velocity level—reference value for the level is set to $v_{0}=1\;\times \;10^{-9}ms$^−1^—clearly shows the resonance points of the beam sample. With increasing frequency, a typical behaviour of plastic is observed, whereby the flexural modulus $E^{\prime }$ and the loss factor η increase non-linearly with the frequency.

### 2.5. Frequency Normalization by Polynomial Interpolation

Due to slight frequency offsets between individual specimens arising from the measurement procedure, the experimentally obtained values of the flexural loss factor η and storage modulus $E^{\prime }$ were not available at identical discrete frequencies for all samples. To ensure comparability across specimens, a frequency normalization procedure was applied prior to the statistical analysis.

For each specimen, the measured frequency-dependent response was approximated by a global polynomial function of third order,

(1) $$y\left(f\right)=a_{0}+a_{1}f+a_{2}f^{2}+a_{3}f^{3},$$

where y(f) denotes either η(f) or $E^{\prime }\left(f\right)$, and *f* is the excitation frequency. The polynomial coefficients were determined by an overdetermined least-squares fit based on resonance points for each specimen, providing inherent smoothing of random measurement noise.

The fitted polynomial was subsequently evaluated at predefined reference frequencies (125 Hz, 250 Hz, 500 Hz, 1000 Hz, 2000 Hz and 4000 Hz), which served as standardized comparison values for all specimens. The interpolation was strictly confined to the experimentally covered frequency range; no extrapolation beyond the outermost measured frequencies was performed.

The use of a global low-order polynomial was chosen as a compromise between robustness against measurement noise and the avoidance of high-frequency oscillations associated with higher-order fits. As the frequency offsets between individual measurements were small relative to the investigated frequency range, the applied interpolation procedure is expected to introduce only minor additional uncertainty while enabling a consistent comparison of frequency-dependent material properties across all specimens.

### 2.6. Statistical Analysis

Statistical analyses were conducted to quantify the influence of the investigated process parameters on the measured response variables. All continuous input factors were z-standardized prior to analysis to ensure numerical stability and comparability of regression coefficients. Replicated specimens were treated as independent observations.

A fixed-effects ordinary least squares (OLS) regression model was formulated, including all main effects, pairwise interaction terms, and quadratic terms of the continuous factors. The statistical significance of individual model terms was evaluated using a Type II analysis of variance (ANOVA). Statistical significance was assessed at a significance level of α=0.05. Corresponding *p*-values represent the probability of observing the given effect, or a more extreme one, under the null hypothesis of no effect. Model terms with p<α were considered statistically significant.

To mitigate the influence of multicollinearity, variance inflation factors (VIFs) were computed for all model terms. Terms exhibiting VIF values greater than 5 were iteratively removed prior to further analysis. Following VIF-based pruning, a hierarchical backward elimination procedure was applied to remove statistically non-significant terms while preserving model hierarchy.

Model diagnostics included the evaluation of residual distributions, normal probability plots, and residuals plotted against fitted values and run order to assess normality, homoscedasticity, and independence of residuals. Influential observations were identified using the difference in fits (DFFITS) criterion with a threshold of $2\sqrt{p/n}$, where *p* denotes the number of model parameters and *n* the number of observations. This threshold follows commonly used diagnostic guidelines to identify observations with a substantial influence on fitted values. Detected outliers were documented but not removed unless stated otherwise.

Model adequacy was further assessed by a lack-of-fit test based on replicated design points, allowing separation of pure experimental error from model residuals. At a significance level of α=0.05, p<α indicates significant lack-of-fit, whereas p≥α indicates an adequate model fit.

All statistical analyses were performed in Python (version 3.12.7, Anaconda distribution) using the statsmodels library (version 0.14.2). Supporting numerical computations were carried out with numpy (version 1.26.4) and scipy (version 1.13.1), while data handling was conducted using pandas (version 2.2.2). Visualization of statistical results was performed with matplotlib (version 3.9.2) and seaborn (version 0.13.2).

## 3. Results

The influence of the investigated process parameters on the acoustic response was evaluated separately for each reference frequency using analysis of variance (ANOVA). For each frequency, a final regression model was obtained after VIF-based term reduction and hierarchical backward elimination. The statistical significance of linear terms, quadratic terms, and interaction terms was assessed using a Type II ANOVA at a significance level of α=0.05.

### 3.1. ANOVA Results for Flexural Storage Modulus $E^{\prime }$

Table 2 summarizes the frequency-resolved significance of linear, interaction, and quadratic terms retained in the final regression models for $E^{\prime }$. While the dominant linear and interaction effects are consistent across all reference frequencies, selected quadratic contributions exhibit frequency-dependent relevance.

Across the investigated reference frequencies, a largely consistent set of statistically significant linear, interaction, and quadratic terms was retained in the final regression models for the flexural storage modulus $E^{\prime }$. Specifically, layer height, printing speed, build plate temperature, and flow rate exhibited significant linear contributions in all shown reference frequencies. Moreover, the two-factor interactions between layer height and printing speed, layer height and flow rate, as well as build plate temperature and flow rate were consistently retained.

Pronounced nonlinear dependencies were captured by significant quadratic contributions of layer height, printing speed, and flow rate throughout the analyzed frequency range. In addition, a quadratic term of build plate temperature was retained at the lower frequencies (125 Hz and 250 Hz), whereas this term was not present in the final models at 500–4000 Hz.

In contrast, extrusion temperature did not exhibit a statistically significant linear contribution in any of the reported reference frequencies and was not retained in the final models after the model reduction procedure. This indicates that, within the investigated parameter range, variations in extrusion temperature did not measurably affect the flexural storage modulus $E^{\prime }$.

### 3.2. Main Effects and Interactions for $E^{\prime }$

To visualize the influence of the statistically significant terms identified by the ANOVA, model-based response curves were evaluated jointly across all investigated reference frequencies using the corresponding final regression models. In this representation, frequency-dependent response curves are overlaid for each process parameter, enabling a direct comparison of frequency-dependent trends.

**Figure 3:**
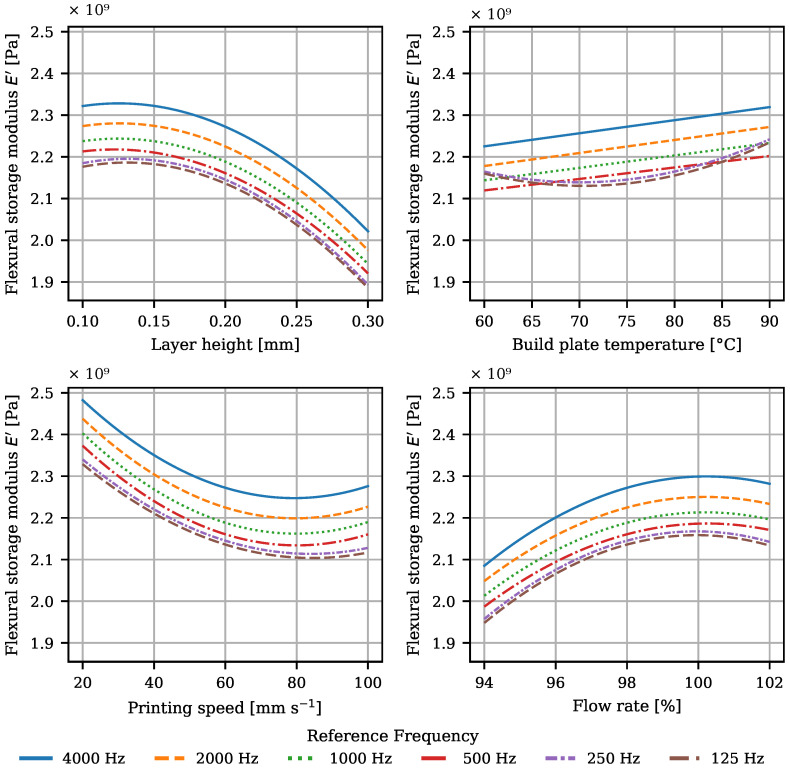
Significant linear and quadratic effects of process parameters on flexural storage modulus $E^{\prime }$ across frequency bands.

Figure 3 summarizes the statistically significant main effects of the investigated process parameters on the flexural storage modulus $E^{\prime }$ across the analyzed reference frequencies. For all parameters, the qualitative trends are largely consistent between frequencies, while the absolute magnitude of $E^{\prime }$ increases systematically with increasing excitation frequency.

Layer height exhibits a pronounced nonlinear dependence. Across all reference frequencies shown, $E^{\prime }$ attains its maximum at low layer heights and decreases continuously with increasing layer height over the investigated range, indicating that higher layer thickness is associated with reduced stiffness.

Build plate temperature shows an overall positive effect on $E^{\prime }$, with higher temperatures generally resulting in higher flexural storage moduli. The trend is approximately linear at higher frequencies, whereas slight curvature is visible at lower frequencies, suggesting a modest frequency-dependent sensitivity.

Printing speed displays a clearly nonlinear dependence: $E^{\prime }$ decreases markedly with increasing speed from low to intermediate values, followed by a shallow minimum and a moderate increase toward the highest speeds.

Similarly, flow rate exhibits a pronounced nonlinear influence on $E^{\prime }$, with increasing modulus toward near-nominal flow conditions and a plateau or slight decrease at the upper end of the investigated flow-rate range.

**Figure 4:**
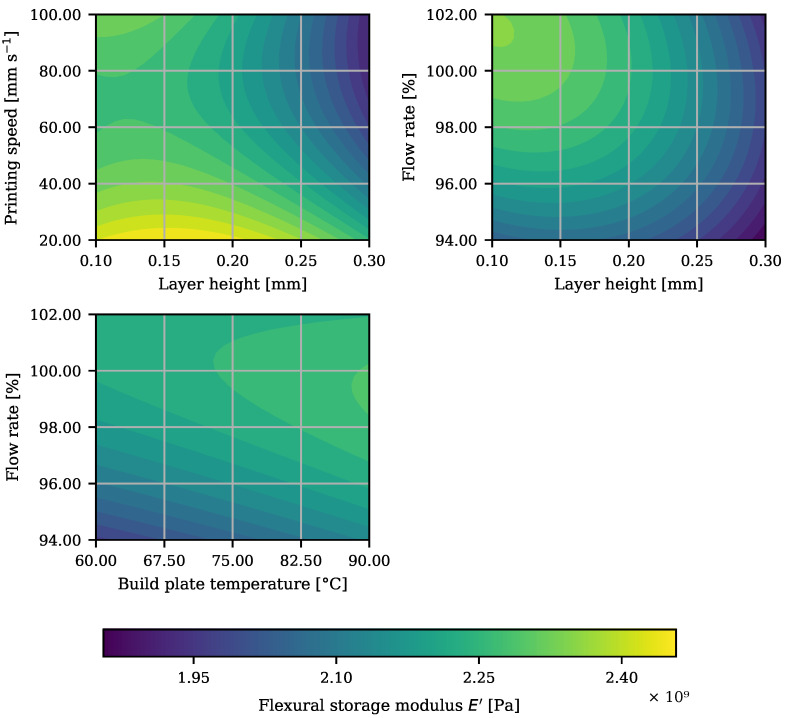
Statistically significant interaction effects for the flexural storage modulus $E^{\prime }$ at 2000 Hz.

Statistically significant interaction effects were identified between layer height and printing speed, layer height and flow rate, as well as build plate temperature and flow rate, as shown in Figure 4. The interaction plots demonstrate that the influence of one process parameter depends on the level of the interacting parameter, particularly at low layer heights and elevated build plate temperatures.

Comparable qualitative trends were observed for the remaining reference frequencies, with differences primarily affecting the magnitude rather than the shape of the main effects and interactions.

### 3.3. Model Adequacy and Residual Analysis for $E^{\prime }$

The adequacy of the frequency-dependent regression models for $E^{\prime }$ was assessed individually for each investigated frequency band. Across all models, consistently high coefficients of determination were obtained ($R^{2}=0.831$–0.847, $R_{\mathrm{adj}}^{2}=0.806$–0.818), indicating a good agreement between experimental data and model predictions.

Residual diagnostics confirmed that the assumptions underlying the Type-II ANOVA were satisfied. Residuals were approximately normally distributed, and no systematic trends were observed with respect to fitted values or run order. Between five and six influential observations were identified using the DFFITS criterion per reference frequencies. However, these observations were retained, as no systematic measurement errors were identified.

Lack-of-fit tests based on pure error estimation revealed no statistically significant lack-of-fit for any investigated reference frequency (p>0.30), confirming that the selected regression models adequately describe the experimental data within the investigated parameter space.

**Figure 5:**
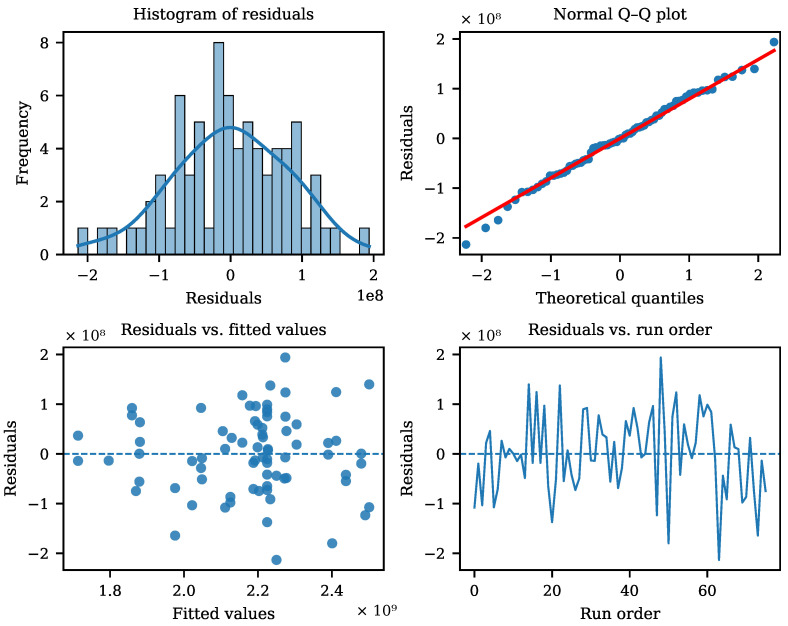
Residual diagnostics for the regression model of $E^{\prime }$ at 2000 Hz. The normal Q–Q plot compares the residuals (blue markers) with the theoretical normal-distribution reference (red line). The histogram includes the fitted normal density (blue curve), while the dashed blue lines indicate zero residuals in the residual plots.

Figure 5 presents the residual diagnostics for the representative reference frequency of 2000 Hz. Corresponding analyses for the remaining reference frequencies yielded comparable results.

### 3.4. ANOVA Results for Loss Factor η

Table 3 provides a compact overview of the statistically significant linear, quadratic, and interaction terms retained in the final regression models for each reference frequency.

Across all investigated frequencies, extrusion temperature exhibited a statistically significant linear contribution to η (p<0.05), indicating a robust frequency-independent sensitivity. In contrast, the relevance of other process parameters varied substantially with frequency.

A pronounced nonlinear dependence on flow rate was observed. The quadratic flow-rate term was statistically significant across a broad frequency range (125–2000 Hz), while the corresponding linear term was not significant, indicating a non-monotonic relationship between flow rate and η within the investigated parameter range.

Interaction effects were detected only in selected reference frequencies, most prominently involving printing speed and flow rate at intermediate frequencies. No statistically significant interactions were identified at 125 Hz, 250 Hz and 4000 Hz.

### 3.5. Main Effects and Interactions for η

**Figure 6:**
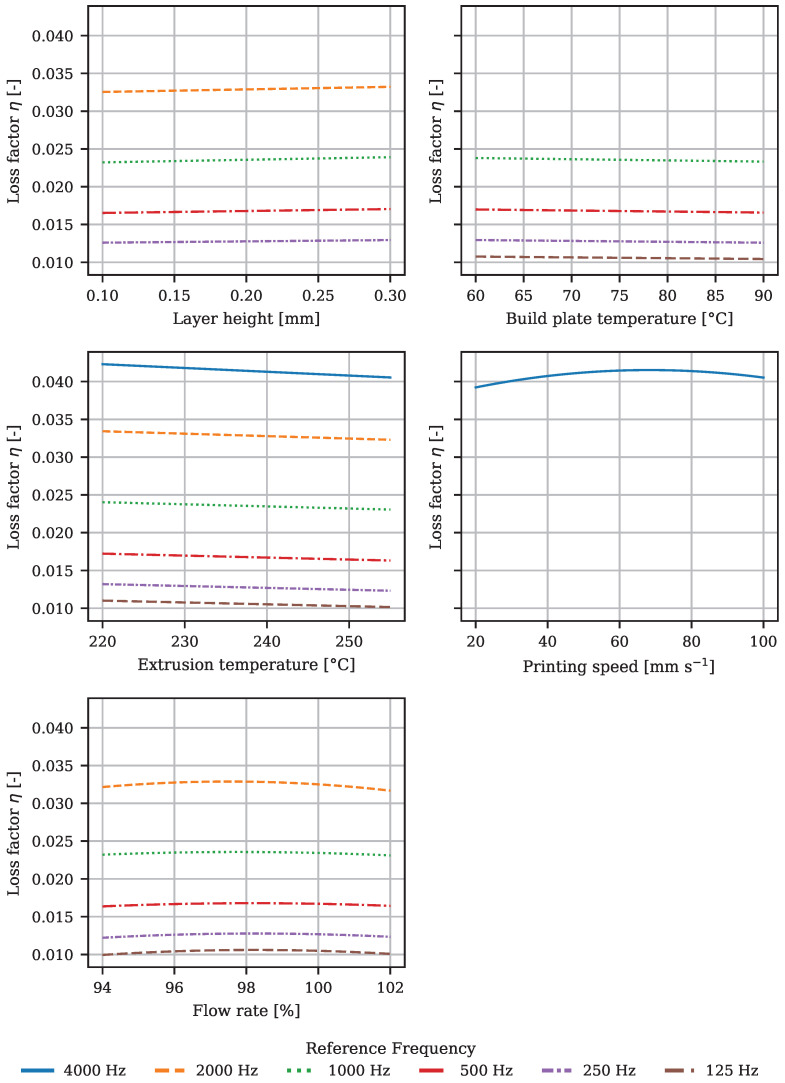
Significant linear and quadratic effects of process parameters on loss factor η across frequency bands.

Figure 6 illustrates the model-based response curves for the statistically significant linear and quadratic terms identified in the ANOVA.

Layer height exhibits statistically significant effects on η in the intermediate frequency range (250–2000 Hz), while no systematic dependence is observed at 4000 Hz. Build plate temperature primarily affects η at low to intermediate frequencies (125–1000 Hz).

Extrusion temperature represents the most consistently influential parameter, contributing significantly to η across all frequency bands. Printing speed, in contrast, exhibits a statistically significant effect only at 4000 Hz.

**Figure 7:**
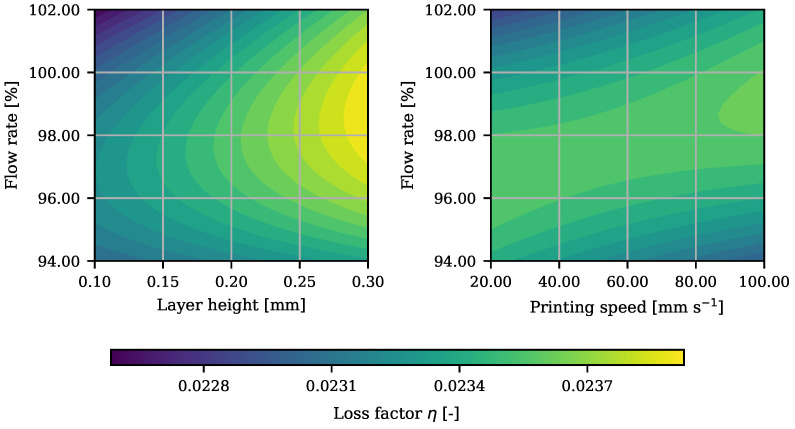
Interaction effects between layer height and flow rate (**left**) as well as printing speed and flow rate (**right**) on the loss factor η at 1000 Hz.

For the loss factor η, interaction effects were observed to be frequency-dependent (Table 3). Among the investigated frequency bands, the model at 1000 Hz was the only case in which multiple interaction terms were retained in the final Type-II ANOVA model. Consequently, interaction plots are shown exemplarily for this frequency band in Figure 7.

The interaction between layer height and flow rate (Figure 7, left) reveals a non-additive influence on η, with the predicted loss factor increasing toward larger layer heights in combination with elevated flow rates. In contrast, at lower flow rates the effect of layer height is less pronounced, indicating a coupled dependence of both parameters.

A similar interaction behavior is observed between printing speed and flow rate (Figure 7, right). At intermediate flow rates, increasing printing speed is associated with higher predicted values of η, whereas this trend diminishes toward lower and higher flow rates. These results confirm that flow rate acts as a moderating parameter for both layer height and printing speed at 1000 Hz.

### 3.6. Model Adequacy and Residual Analysis for η

The adequacy of the frequency-dependent regression models for the loss factor η was evaluated using goodness-of-fit metrics and residual diagnostics. An overview of the model complexity, explanatory power, and global significance for each investigated frequency band is provided in Table 4.

All regression models were statistically significant according to the global *F*-test (p<0.01). The adjusted coefficient of determination varied markedly with frequency, ranging from $R_{\mathrm{adj}}^{2}=0.215$ at 4000 Hz to $R_{\mathrm{adj}}^{2}=0.763$ at 500 Hz, indicating a pronounced frequency dependence of the model explanatory power. In particular, the comparatively low explanatory power at 4000 Hz suggests that a substantial fraction of the variability in η at high frequencies is not captured by the investigated process parameters. Normality of the residuals was assessed using the Jarque–Bera test. For five out of six frequencies, no statistically significant deviations from normality were detected. In contrast, the regression model at 4000 Hz exhibited a significant Jarque–Bera test result ($p_{\mathrm{JB}}=0.003$), indicating non-normal residuals.

Residual independence was evaluated using the Durbin–Watson statistic. The obtained values for DW ranged from 1.61 to 2.00 across all frequencies, indicating no pronounced residual autocorrelation. Lack-of-fit tests further confirmed an adequate model structure for five frequencies. For the model at 500 Hz, however, a statistically significant lack-of-fit was observed ($p_{\mathrm{LoF}}=0.049$), suggesting that the selected regression model does not fully capture the observed variability of η at this frequency.

Influential observations were identified using the DFFITS criterion. Depending on frequency, up to four observations exceeded the threshold value. These observations were retained in the analysis, as no systematic experimental or measurement-related anomalies were identified.

## 4. Discussion

This study investigated how key material-extrusion process parameters influence the frequency-dependent flexural storage modulus $E^{\prime }$ and loss factor η of additively manufactured specimens. The results reveal a systematic separation between parameters governing stiffness-related response ($E^{\prime }$) and those primarily affecting energy dissipation (η), as well as a pronounced difference in how robustly these responses can be captured by frequency-resolved regression models within the investigated design space.

### 4.1. Contrasting Frequency Dependence of $E^{\prime }$ and η

A central finding is that the final model structure for $E^{\prime }$ remained invariant across all investigated frequency bands, whereas the model structure for η varied substantially with frequency. This contrast suggests that, within the explored parameter range, the dominant process-induced factors affecting stiffness act in a largely frequency-independent manner, while dissipation-related mechanisms are more sensitive to frequency-dependent microstructural and interfacial phenomena. A plausible explanation is that dissipation is governed by viscoelastic relaxation processes and interfacial friction at strand boundaries, both of which are known to exhibit characteristic time and frequency dependencies in thermoplastic polymers.

The consistently high coefficients of determination for $E^{\prime }$ across all bands further support the interpretation that the selected process parameters capture the major drivers of the stiffness response. In contrast, the wider spread in $R^{2}$ and $R_{\mathrm{adj}}^{2}$ values for η indicates that, although statistically significant and physically meaningful effects were detected, the loss factor is influenced by additional variability not fully explained by the selected predictors or by the adopted polynomial model structure at every frequency.

### 4.2. Process Parameters Governing $E^{\prime }$: Robust Effects and Nonlinearities

For the flexural storage modulus, layer height, printing speed, build plate temperature, and flow rate consistently contributed as statistically significant linear terms, accompanied by significant quadratic terms for layer height, printing speed, and flow rate. These process parameters have been widely recognized in the additive manufacturing literature to influence mechanical properties through their effects on interlayer adhesion, porosity, and defect formation in MEX parts [4,5]. While these studies predominantly address static mechanical properties, the underlying process–structure relationships they describe are equally relevant for stiffness-dominated responses probed under dynamic loading, such as the storage modulus $E^{\prime }$. The observed increase in $E^{\prime }$ with excitation frequency is further consistent with previous dynamic mechanical investigations of PETG-based systems reported in the literature [13]. However, beyond the frequency dependence of the modulus itself, the consistent retention of the same linear, quadratic, and interaction effects across all investigated frequencies indicates that the governing process–structure relationships for $E^{\prime }$ remain stable within the studied design space.

The nonlinear dependence on layer height and flow rate suggests that stiffness is not governed by monotonic trends alone, but rather by an interplay of factors that may include geometric discretization, interlayer contact area, and process-dependent material placement. The presence of quadratic terms indicates that both too low and too high settings can move the response away from an optimum region, which is consistent with the observed non-monotonic main-effect shapes.

In addition, significant interactions between layer height and printing speed, layer height and flow rate, and build plate temperature and flow rate highlight that extrusion-related parameters cannot be treated independently. In statistically designed experiments, varying multiple factors simultaneously enables estimation of interaction effects that would be overlooked in one-factor-at-a-time approaches [16]. Instead, the stiffness response emerges from coupled process conditions, implying that optimization strategies should consider combined parameter settings rather than one-factor-at-a-time adjustments. Importantly, these interactions were retained across all frequencies, reinforcing their relevance as systematic contributors rather than frequency-specific artifacts.

Notably, extrusion temperature did not contribute significantly to $E^{\prime }$ in any frequency band. Within the investigated range, this indicates that changes in extrusion temperature did not measurably affect the stiffness response once other parameters were accounted for. This finding suggests that, for the material and conditions considered, stiffness is primarily controlled by geometric and deposition-related variables rather than by temperature-driven changes captured by the present model terms. These observations are specific to PETG within the investigated parameter range and may not directly transfer to semi-crystalline or fiber-reinforced polymer systems, where additional microstructural mechanisms may dominate.

### 4.3. Determinants of η: Frequency-Dependent Sensitivities and Nonlinear Flow-Rate Effects

Compared to $E^{\prime }$, the loss factor η exhibited markedly frequency-dependent sensitivities. Extrusion temperature was consistently retained as a statistically significant linear term across all frequency bands, indicating that temperature-dependent effects systematically influence dissipation. In contrast, the significance of layer height and build plate temperature was limited to specific frequency ranges, and interaction terms appeared only in selected bands. This pattern suggests that the observed frequency dependence of η can be attributed to dissipation mechanisms that are inherently sensitive to excitation frequency, most notably viscoelastic losses, as commonly described in dynamic mechanical analysis [8], with additional contributions potentially arising from interfacial friction and structural heterogeneity.

Frequency-dependent viscoelastic behavior in additively manufactured polymers has been reported in previous dynamic mechanical studies. For instance, Domingo-Espín et al. [17] demonstrated that MEX-printed polymers exhibit pronounced changes in storage and loss-related properties as a function of excitation frequency and temperature under dynamic loading. Similarly, Liu et al. [18] reported significant frequency-dependent variations in the viscoelastic response of additively manufactured polymer systems, indicating that dynamic properties cannot be inferred directly from static mechanical performance alone. However, these studies primarily focused on specific materials or individual processing conditions and did not systematically relate frequency-dependent responses to a multivariate MEX process parameter space using statistically rigorous regression models.

A key result for η is the persistent significance of the quadratic flow-rate term across most frequencies, combined with the absence of a retained linear flow-rate term. Statistically, this indicates that the primary influence of flow rate on η is governed by curvature within the investigated range rather than by a monotonic trend. Practically, this implies the existence of an intermediate flow-rate region associated with increased or decreased damping relative to the extremes, depending on frequency and interaction context, and potentially reflecting competing effects of strand compaction, interlayer contact conditions, and defect formation as the material feed deviates from under- or over-extrusion regimes. The recurring nonlinearity of flow rate also provides a plausible explanation for why simple linear process heuristics may be insufficient to control damping behavior.

Interaction effects for η were restricted to intermediate frequencies, predominantly involving the coupling between printing speed and flow rate, and additionally between layer height and flow rate at 1000 Hz. The selective appearance of these interaction terms may indicate that, within the investigated frequency range, dissipation is particularly sensitive to combined changes in deposition kinematics and material feed at intermediate excitation frequencies, although the underlying physical origin of this frequency selectivity cannot be unambiguously resolved within the present dataset. From a process-control perspective, these results suggest that parameter coupling is most critical in the intermediate frequency regime, whereas at very low and very high frequencies, the retained effects are primarily driven by a smaller subset of terms.

### 4.4. Model Adequacy: Implications for Interpretability and Limitations

Model diagnostics showed that the regression models for $E^{\prime }$ satisfy standard assumptions and exhibit no statistically significant lack-of-fit across the investigated bands. This supports the use of the fitted models for describing trends and for identifying robust process sensitivities.

For η, residual diagnostics were acceptable for most bands, but two limitations require explicit consideration. First, the 4000 Hz model exhibited significant deviations from normality, accompanied by positive skewness and increased kurtosis. This indicates that inference based on normality assumptions should be interpreted with caution for this band. Although the model remains statistically significant, the residual distribution suggests that unmodeled effects or non-Gaussian measurement variability may become more influential at high frequencies.

Second, the statistically significant lack-of-fit observed at 500 Hz indicates that the selected polynomial model structure does not fully capture the variability in η at this frequency. This does not invalidate the detected significant terms, but it limits the reliability of point-wise predictions and suggests that additional model flexibility or additional predictors may be required to better represent the underlying behavior at this band. Potential remedies include higher-order terms, alternative functional forms, or the inclusion of additional process- or specimen-level descriptors, provided that the resulting model complexity remains justified relative to the available data and replication.

Finally, influential observations identified by DFFITS were retained because no systematic measurement errors were identified. While this choice preserves the integrity of the experimental dataset, it may contribute to reduced normality or localized lack-of-fit in certain bands. Future work could evaluate robustness by comparing model structures and key effects with and without influential observations as a sensitivity analysis, while keeping the primary conclusions grounded in the full dataset.

### 4.5. Implications for Process Optimization and Design of Damping-Critical Structures

Taken together, the results indicate that stiffness ($E^{\prime }$) can be tuned reliably through a consistent set of process parameters across frequencies, whereas damping (η) requires frequency-aware optimization due to its stronger sensitivity to parameter selection and coupling. In practical terms, this implies that process settings optimized for maximizing stiffness may not coincide with settings that maximize damping, and that multi-objective, model-based optimization strategies should explicitly incorporate the target frequency range of the intended application.

The observed nonlinearities and interactions, particularly those involving flow rate, further suggest that parameter optimization for damping-critical components should focus on identifying stable operating regions rather than extrapolating from linear trends. Given the frequency dependence of η, it may be advantageous to define application-specific objective functions (e.g., band-averaged damping) and to validate predicted optima experimentally, especially in bands where diagnostics indicate reduced adequacy.

### 4.6. Summary of Key Findings

In summary, $E^{\prime }$ was governed by a frequency-invariant model structure with consistently strong fits and stable parameter effects, whereas η exhibited pronounced frequency dependence with consistent sensitivity to extrusion temperature and a robust nonlinear dependence on flow rate. Interaction terms for η were limited to intermediate frequencies, and diagnostic results highlighted specific frequency bands where caution is warranted in interpretation (notably 4000 Hz for normality and 500 Hz for lack-of-fit). These findings provide a quantitative basis for frequency-aware process tuning within the investigated design space in additively manufactured components where both stiffness and damping are performance-critical.

## 5. Conclusions

This work demonstrates that stiffness- and damping-related responses in additively manufactured structures are governed by distinct process sensitivities and exhibit different levels of frequency dependence. By combining frequency-resolved regression modeling with systematic term reduction and diagnostic assessment, the study provides a coherent framework for separating robust, frequency-invariant effects from frequency-specific sensitivities within a unified experimental design. The reported findings are specific to PETG within the investigated process window, printing conditions, and frequency range up to 4000 Hz, and may not be directly transferable to other polymer systems or infill configurations without further validation.

The flexural storage modulus $E^{\prime }$ was shown to be well described by a stable and invariant model structure across all investigated frequency bands. This robustness indicates that, within the explored parameter space, stiffness can be predictably tailored using a consistent set of process parameters, supporting the transferability of optimized settings across a wide frequency range.

In contrast, the loss factor η required frequency-dependent model structures, reflecting the inherently more complex and variable nature of dissipation-related mechanisms. The results highlight extrusion temperature as a consistently influential parameter and reveal a dominant nonlinear contribution of flow rate, underscoring that damping behavior cannot be controlled reliably through linear parameter adjustments alone. The selective occurrence of interaction effects further emphasizes that damping optimization must account for coupled process conditions and target-specific frequency ranges.

From an application perspective, these findings imply that process settings optimized for stiffness are not necessarily suitable for controlling damping, particularly in frequency-critical designs. Consequently, additive manufacturing strategies for vibration-sensitive components should adopt frequency-aware and multi-objective optimization approaches rather than relying on globally optimal parameter sets.

Overall, this study establishes a statistically rigorous basis for distinguishing stiffness- and damping-driven process effects in material extrusion and provides actionable guidance for the design and optimization of additively manufactured structures where both mechanical rigidity and energy dissipation are performance-critical. Future work may extend this framework to other polymer systems, broader frequency ranges, or alternative modeling approaches to further elucidate dissipation mechanisms in material extrusion.

## Figures and Tables

**Table 1:** Investigated process parameters and factor levels for DOE 1.

Factor	Unit	Levels		
		−1	0	+1
layer height	mm	0.1	0.2	0.3
printing speed	mm/s	20	60	100
extrusion temperature	°C	220	237.5	255
build plate temperature	°C	60	75	90
flow rate	%	94	98	102

**Table 2:** Overview of statistically significant effects retained in the final Type II ANOVA models for the flexural storage modulus $E^{\prime }$ across all investigated reference frequencies. Significance levels are indicated according to the corresponding *p*-values.

Model Term	125 Hz	250 Hz	500 Hz	1000 Hz	2000 Hz	4000 Hz
Layer height (linear)	***	***	***	***	***	***
Build plate temperature (linear)	*	*	**	**	**	**
Printing speed (linear)	***	***	***	***	***	***
Flow rate (linear)	***	***	***	***	***	***
Layer height × printing speed	***	***	***	***	***	***
Layer height × flow rate	***	***	***	***	***	***
Build plate temperature × flow rate	*	*	*	*	*	*
Layer height (quadratic)	**	**	**	**	**	**
Build plate temperature (quadratic)	*	*	–	–	–	–
Printing speed (quadratic)	*	*	**	**	**	**
Flow rate (quadratic)	**	**	*	*	*	*

Significance levels: *** denotes p<0.001, ** denotes 0.001≤p<0.01, * denotes 0.01≤p<0.05, and – denotes not significant.

**Table 3:** Overview of statistically significant effects retained in the final Type-II ANOVA models for the loss factor η across all investigated reference frequencies. Significance levels are indicated according to the corresponding *p*-values.

Model Term	125 Hz	250 Hz	500 Hz	1000 Hz	2000 Hz	4000 Hz
Layer height (linear)	–	**	***	***	**	–
Build plate temperature (linear)	*	**	***	**	–	–
Extrusion temperature (linear)	***	***	***	***	***	*
Printing speed (linear)	–	–	–	–	–	*
Flow rate (linear)	–	–	–	–	–	–
Layer height × flow rate	–	–	–	*	–	–
Printing speed × flow rate	–	–	**	**	*	–
Flow rate (quadratic)	**	***	***	**	***	–
Printing speed (quadratic)	–	–	–	–	–	*

Significance levels: *** denotes p<0.001, ** denotes 0.001≤p<0.01, * denotes 0.01≤p<0.05, and – denotes not significant.

**Table 4:** Goodness-of-fit and residual diagnostics for the frequency-dependent regression models of the loss factor η.

*f* [Hz]	$R_{\mathrm{adj}}^{2}$	p(F)	$p_{\mathrm{JB}}$	DW	$p_{\mathrm{LoF}}$	DFFITS
125	0.599	$2.37\times 10^{-8}$	0.713	1.61	0.890	2
250	0.748	$1.04\times 10^{-11}$	0.869	1.90	0.704	4
500	0.763	$3.50\times 10^{-11}$	0.536	2.00	0.049	2
1000	0.685	$1.44\times 10^{-8}$	0.875	1.80	0.253	3
2000	0.595	$2.03\times 10^{-7}$	0.223	1.89	0.313	0
4000	0.215	$4.66\times 10^{-3}$	0.003	1.85	0.786	3

## Data Availability

The dataset will be made available on request from the authors.

## Abbreviations

The following abbreviations are used in this manuscript:

ANOVA	analysis of variance
DMA	dynamic mechanical analysis
DFFITS	difference in fits
DOE	design of experiments
E′	flexural storage modulus
FCCD	face-centered composite design
MEX	material extrusion
OLS	ordinary least squares
PETG	polyethylene terephthalate glycol
VIF	variance inflation factor

## References

[1] (2021). Additive Manufacturing-General Principles-Fundamentals and Vocabulary.

[2] Gibson I., Rosen D., Stucker B., Khorasani M. (2021). Additive Manufacturing Technologies.

[3] Cojocaru V., Frunzaverde D., Miclosina C.O., Marginean G. (2022). The Influence of the Process Parameters on the Mechanical Properties of PLA Specimens Produced by Fused Filament Fabrication—A Review. Polymers.

[4] Lou X., Tang X., Dong L., Zhao T., Wang F., Zhao L., Zhang T. (2025). Influence of layer thickness and extrusion ratio on strand morphology, porosity, surface roughness, and anisotropic mechanical properties in FDM. Sci. Rep..

[5] Khan S., Joshi K., Deshmukh S. (2022). A comprehensive review on effect of printing parameters on mechanical properties of FDM printed parts. Mater. Today Proc..

[6] (2019). Plastics-Determination of Dynamic Mechanical Properties—Part 1: General Principles.

[7] (2021). Plastics-Determination of Dynamic Mechanical Properties-Part 3: Flexural Vibration-Resonance-Curve Method.

[8] Menard K.P. (2008). Dynamic Mechanical Analysis—A Practical Introduction.

[9] Rotermund A., Langer S.C., Hoffmann S., Heck P. Vergleich der Verfahren in ISO 6721-3 zur Bestimmung dynamisch-mechanischer Eigenschaften von Kunststoffen. Proceedings of the Fortschritte der Akustik-DAGA 2023.

[10] Rothe S., Blech C., Watschke H., Vietor T., Langer S.C. (2020). Material Parameter Identification for Acoustic Simulation of Additively Manufactured Structures. Materials.

[11] Rothe S., Blech C., Langer S.C., Watschke H., Vietor T. (2019). Layer-effect by additive manufacturing of acoustic black holes. Proceedings of Inter-Noise.

[12] Heck P.M., Rotermund A., Langer S.C. Characterisation methods of the viscoelastic properties of plastics in multi-material designs. Proceedings of the Fortschritte der Akustik-DAGA 2024.

[13] Colón Quintana J.L., Tomlinson S., Lopez-Anido R.A. (2024). Thermomechanical and Viscoelastic Characterization of Continuous GF/PETG Tape for Extreme Environment Applications. J. Compos. Sci..

[14] Ulkir O., Kuncan F., Alay F.D. (2025). Experimental Study and ANN Development for Modeling Tensile and Surface Quality of Fiber-Reinforced Nylon Composites. Polymers.

[15] (2005). Damping of Materials and Members-Experimental Techniques for the Determination of Damping Characteristics.

[16] Czitrom V. (1999). One-Factor-at-a-Time Versus Designed Experiments. Am. Stat. Assoc..

[17] Domingo-Espin M., Borros S., Agullo N., Garcia-Granada A.A., Reyes G. (2014). Influence of Building Parameters on the Dynamic Mechanical Properties of Polycarbonate Fused Deposition Modeling Parts. 3D Print. Addit. Manuf..

[18] Liu G., Hu N., Huang J., Tu Q., Xu F. (2024). Experimental Investigation on the Mechanical and Dynamic Thermomechanical Properties of Polyether Ether Ketone Based on Fused Deposition Modeling. Polymers.

